# Measles outbreak: a warning sign of troubles ahead

**DOI:** 10.3325/cmj.2019.60.393

**Published:** 2019-10

**Authors:** Bernard Kaić, Goran Tešović

**Affiliations:** 1 Croatian Institute of Public Health, Zagreb, Croatia *bernard.kaic@hzjz.hr*; 2 University Hospital for Infectious Diseases, University of Zagreb School of Medicine, Zagreb, Croatia

Measles is a serious and highly contagious infectious disease caused by a paramyxovirus (measles virus, MV) that can easily spread through airborne respiratory droplets or by direct contact with upper respiratory tract secretions of infected individuals (1,2). Following an incubation period of 10-14 days, infected patients experience a fever, coryza or conjunctivitis, cough, and a generalized rash that lasts 5-6 days and spreads in cranio-caudal direction. The disease in the vast majority of patients terminates with pathogen elimination and results in lifelong specific immunity that prevents reinfection (1,3). Although the majority of patients recover without any complication, approximately 30% of cases are complicated by diarrhea, pneumonia, acute otitis media, encephalitis, and even death. Infants and preschool aged children, as well as adults older than 20 years, are more prone to an unfavorable disease course (3). In 0.01% of patients who acquired natural measles infection, MV remains in a dormant form within the neural tissues and progresses, usually after a latency period of 5-10 years, into subacute sclerosing panencephalitis (SSPE) (4-6). SSPE is a chronic devastating encephalitis most commonly affecting children in whom wild MV infection occurred before the age of 2 years (4-7). Although partial and temporary remission could occur, the disease is invariably fatal. Progressive mental and motor deterioration leads to a vegetative state and fatal outcome usually 1-3 years after the occurrence of the first symptoms (4-7). Currently, there is no effective antiviral treatment either for the acute disease or SSPE. Active immunization thus remains the only way for disease burden reduction.

In the pre-vaccination era, more than 90% of children in the world under the age of 15 years were infected with MV, resulting in more than 2 million deaths annually (8). With the introduction of active immunization, the global number of measles cases decreased significantly. During 2000–2011 period, annual measles incidence decreased by 65% worldwide, from 146 to 52 cases per 1 million population, and the estimated number of measles deaths decreased by 71%, from 548 000 to 158 000 (9).

The European Vaccination Action Plan for 2015-2020, based on the World Health Organization (WHO) Global Vaccination Action Plan, anticipates the elimination of measles in the WHO European Region (10). According to this Plan, the interruption of indigenous measles transmission should have been achieved by 2015 and elimination in the WHO European Region should have been declared in 2018. However, continuous transmission with occasional measles outbreaks in the European region still occurs. In the first six months of 2019, approximately 90 000 measles cases were reported in the European Region, which is more than the number reported during the entire 2018 (11). The question arises as to the reasons for the failure to eliminate the disease. From the medical point of view, the prerequisites for elimination and eventual eradication of measles have been met:

• humans are the only natural reservoir of MV,

• long-term carriage and shedding of MV does not occur,

• there is no antigenic diversity among strains and genotypes, so the available vaccines provide protection against all circulating strains of MV,

• the low price of vaccine allows it to be universally available, and

• the vaccine provides long-term protection in a high percentage of vaccinated persons.

Therefore, elimination goals have been unmet not because of the suboptimal characteristics of the available vaccines or disease features, but because of the “logistical” failure of the health systems to achieve optimal vaccination coverage of the European population for a sufficiently long period.

Within the Region, reasons for low vaccination coverage vary. Some countries, in the past two to three decades have faced immunization coverage gaps created by massive migrations, difficulties in vaccine supply, unequitable access to vaccine of particular population subgroups, and breaches in the cold chain affecting vaccine quality. Nevertheless, countries without vaccine supply problems and with good access to vaccination for the majority of the population have also faced inadequate coverage, largely due to the lack of awareness of the need for vaccination, fear of adverse events, which results in delayed or missed vaccinations, or refusal of vaccination by anti-science vaccine opponents (12-14).

Since the introduction of measles vaccination into the Croatian Immunization Program in 1968, Croatia has never experienced a shortage of measles-containing vaccine that would affect vaccine coverage. In the last fifty years, vaccination rates in Croatia have been high enough to ensure a level of population immunity sufficient to interrupt indigenous circulation of the MV in the population and to eliminate measles.

Individual cases of measles are regularly imported to Croatia by Croatian citizens returning from abroad or by foreigners. In the last two decades, the majority of measles importations have not resulted in transmission to contacts due to a high level of population immunity. For example, in 2018 there were eight imported measles cases across Croatia. Only one of these imported cases led to disease transmission to contacts, resulting in a small outbreak in Dubrovnik and the surrounding area (15 directly linked cases). However, as the last year's outbreak in Dubrovnik and this year's clusters of measles in Split, Slavonski Brod, and Zagreb demonstrate, vaccination coverage achieved in the previous decades is not sufficient to completely prevent the circulation of the introduced MV and the occurrence of measles clusters in adults. It is important to emphasize, however, that each of the clusters could have grown to larger outbreaks if timely and extensive outbreak response activities had not been carried out. Nevertheless, the measles outbreaks witnessed in Croatia in the last ten years have been less intensive than the outbreaks in other European countries, such as North Macedonia, Serbia, Italy, France, Romania, or Ukraine, indicating a relatively high level of population immunity in Croatia.

Additionally, we are worried about a negative trend in vaccine coverage in children that occurred over the last eight years. From 2011 to 2017, childhood vaccination rates in Croatia declined steadily. So far, we have been lucky not to have measles introduced into a poorly vaccinated population of preschool children, but in the near future this scenario cannot be ruled out. If vaccination rates continue to decline, even if we are spared the outbreaks in preschool children, the proportion of the susceptible individuals in the population will increase and we can expect major measles outbreaks among children and adolescents.

Measles is not the only potential problem caused by declining vaccination rates. When vaccine coverage decreases, measles is the first disease to resurge only because it is the most contagious of all vaccine-preventable diseases. Although measles can cause serious morbidity with severe complications, including death, as vaccination rates decline, more serious vaccine-preventable diseases may find their way into the population. Therefore, measles outbreaks are a warning sign that we might expect the outbreaks of serious illnesses, such as whooping cough, diphtheria, or polio.

In order to prevent future outbreaks of measles and other vaccine-preventable diseases, we should remove obstacles to vaccination. As noted earlier, barriers to vaccination in Croatia are not the lack of vaccines and inadequate storage and transportation conditions, but a lack of interest in vaccination, fear of vaccinations, and anti-scientific attitudes. These barriers are not only present among lay people, who perpetuate unscientific attitudes through social networks, but also among health care professionals, who, due to lack of confidence, attribute false contraindications to vaccination and unnecessarily delay vaccinations, further contributing to parents’ skepticism. In Croatia, as in most European countries, research has shown that, despite the abundance of information available on the internet and social networks, health care workers are the major source of information and have the greatest influence on the attitudes of young parents toward childhood vaccinations (15-19). Therefore, vaccine coverage can be considerably increased by empowering the attitudes, skills, and knowledge of primary care physicians through better, science-based, education of physicians about the benefits, risks, purpose, and procedures of vaccination. Of course, there will always be a small number of individuals who refuse to accept global scientific consensus. However, many parents who hesitate to vaccinate their children might overcome their fear or lack of recognition of the need for vaccination if they see the primary care physician’s positive attitude toward vaccination based on a thorough understanding of the benefits and risks of vaccination.

The medical profession can contribute to the ongoing debate on compulsory vaccination in Croatia with three evidence-based pieces of information:

1. It is in the interest of the protection of individuals, public health, and economic prosperity of the population, to continuously maintain high vaccine coverage in the population, as this will eliminate some vaccine-preventable diseases and curb the spread of the diseases that cannot be eliminated, reducing the suffering, treatment expenditure, and loss of life.

2. In European countries, no correlation has been established between the legal status of vaccination and coverage rates (20,21). Some countries where childhood vaccinations are strongly recommended but not mandatory, such as the Scandinavian countries, have higher vaccination coverage than countries with statutory vaccination. Countries that mandate vaccination against some diseases and only recommended it against others achieve higher coverage for mandatory vaccinations. For example, in France a higher coverage was achieved for the compulsory polio vaccine than for the recommended measles vaccine. Measles vaccination in France became mandatory in 2018, but it is still too early to assess the impact of this legislative change on vaccination coverage.

3. A correlation has been observed between vaccination rates and the involvement of the attending physicians and the availability of services. Countries where physicians are more diligent in calling and reminding parents about scheduled and missed childhood vaccinations achieve higher coverage rates (22,23).
